# *Olea europaea* L. (olive) leaf extract ameliorates learning and memory deficits in streptozotocin-induced diabetic rats 

**DOI:** 10.22038/AJP.2021.18989

**Published:** 2022

**Authors:** Ali Akbar Asghari, Mahmoud Hosseini, Soleyman Bafadam, Hasan Rakhshandeh, Maryam Farazandeh, Maryam Mahmoudabady

**Affiliations:** 1 *Department of Physiology, Faculty of Medicine, Mashhad University of Medical Sciences, Mashhad, Iran*; 2 *Division of Neurocognitive Sciences, Psychiatry and Behavioral Sciences Research Center, Mashhad University of Medical Sciences, Mashhad, Iran*; 3 *Pharmacological Research Center of Medicinal Plants, Mashhad University of Medical Sciences, Mashhad. Iran*; 4 *Applied Biomedical Research Center, Mashhad University of Medical Sciences, Mashhad, Iran*

**Keywords:** Diabetes, Oxidative stress, Memory, Olive leaf extract

## Abstract

**Objective:**

The aim of the present study was to assess olive leaf extract (OLE) effects on learning and memory deficits in a model of diabetes induced by streptozotocin (STZ) in rats.

**Materials and methods:**

The rats were divided as: (1) control rats, (2) diabetic rats, and (3–6) diabetic rats treated by 100, 200, and 400 mg/kg of OLE or metformin. Using the passive avoidance test (PA), we investigated fear learning and memory behaviors. In cortical and hippocampus tissues, total levels of malondialdehyde (MDA) and thiol were measured along with the activity of catalase (CAT) and superoxide dismutase (SOD).

**Results:**

Learning and memory behavior impairment were significantly developed in diabetic rats as shown by the impairment of the PA task compared to the control group (p<0.001). In addition, elevated levels of MDA and reduced overall concentrations of thiol, CAT and SOD activity were obvious in diabetic rats’ cortex and hippocampus tissues (p<0.01–p<0.001). Meanwhile, OLE in a dose-dependent manner, improved memory deficit and cognitive performance that was attributed to a reduction of lipid peroxidation and elevation of total thiol concentration, and CAT and SOD activity levels in the brain tissues (p<0.05–p<0.001).

**Conclusion:**

OLE could be effective in improving cognitive impairment in STZ-induced diabetes by oxidative stress depression.

## Introduction

The human brain is a complicated system and numerous factors, such as age and different illnesses, can influence its function (Kumar et al., 2010 ▶). Diabetes mellitus (DM) is related to a number of acute and chronic problems in various organs, and it affects the central nervous system and causes cognitive impairment (Liu et al., 2016 ▶). Although diabetes has an important role in memory impairment, the mechanisms that induce memory and learning deficit, remain unclear (Moghadamnia et al., 2015 ▶). It is stated that variables such as decreased neuronal densities, oxidative stress, and metabolic impairments are essential in the pathogenesis of memory deficit and cognitive impairment (Hasanein and Shahidi, 2010 ▶). In diabetes condition, hyperglycemia increases glucose oxidation, which generates reactive oxygen species (ROS) and induces oxidative stress (Modi et al., 2015 ▶). Oxidative imbalance like excess malondialdehyde (MDA) and decreased catalase (CAT) activity leads to morphological and functional modifications in the cortical regions and hippocampus (Good, 2002 ▶).

The olive tree (*Olea europaea* L.) of the Oleaceae family is grown in many countries, particularly, in the Mediterranean region (Ryan and Robards, 1998 ▶; Somova et al., 2003 ▶). *Olea europaea*, has been considered a plant with a distinctive antioxidant ability in its fruits, leaves and oil (Briante et al., 2002 ▶; Gerber, 1994 ▶; Soni et al., 2006 ▶). One of the byproducts extracted from the olive leaves are phenolic compounds that have beneficial antioxidants properties (Andreadou et al., 2007 ▶). Oleuropein, hydroxytyrosol, luteolin, and apigenin are the phenolic compounds found in olive leaf extract (OLE). The most abundant phenolic compound already explored in olive leaf extract is oleuropein (Al-Azzawie and Alhamdani, 2006b ▶; Benavente-Garcıa et al., 2000 ▶). Experimental studies recognized the valuable impacts of oleuropein and its products such as hydroxytyrosol including stimulating antioxidant enzymes (Andreadou et al., 2006 ▶), immunoregulatory effects (Visioli et al., 1998 ▶), anticancer and chemopreventive activities (Hamdi and Castellon, 2005 ▶), anti-hypoglycemic activity (Jemai et al., 2009 ▶), and inhibition of platelet aggregation (Manna et al., 2004 ▶); it also has beneficial effects against viruses and bacteria (Bisignano et al., 1999 ▶), and neurodegenerative diseases (Bazoti et al., 2006 ▶). In this regard, a study has shown that oleuropein has neuroprotective effects in the hippocampus on memory impairment in colchicine-induced rat model of Alzheimer's disease (Pourkhodadad et al., 2016 ▶). Several phenolic components especially oleuropein, have been considered to have interaction with different amyloidogenic proteins to reduce amyloid information (Leri and Bucciantini, 2016 ▶). Regarding valuable and beneficial anti-oxidative and anti-inflammatory properties of OLE and its constituents, we aimed to investigate the effect of long-term oral administration of OLE on learning and memory deficits in diabetic rats using passive avoidance test. The oxidative stress status was also measured by assessment of biomarkers such as MDA, total thiol concentration, and CAT and superoxide dismutase (SOD) activity in the hippocampal and cortical regions.

## Materials and Methods

**Characterization and Preparation of the plant extract**

Olive leaves were collected from Bardaskan City, Khorasan Province, Iran in early summer 2019 when the plant wildly grows in the normal condition; the plant was identified and a voucher number was dedicated by a botanist (E 1253 (FUMH). According to the protocol, to prepare hydroethanolic extract, dried leaves of the plant were completely macerated in ethanol (50%) for three days. The acquired extract was filtrated and consequently dried using a rotary vacuum evaporator. The yield of dried extract related to the weight of the dried leafs, was 18%. To acquire varying doses (100, 200 and 400 mg/kg) of OLE, it was dissolved in distilled water.

**Animals and diabetes induction**

Sixty male Wistar rats, 10 weeks of age, 250-300 g, were maintained at room temperature of 22±2°C under 12 hr light/dark cycles with unlimited access to chow and water. 

The study was authorized by Mashhad University of Medical Sciences, Mashhad, Iran, Committee on Animals Research (approval No: IR.MUMS.MEDICAL.REC.1397.739).

Streptozotocin (STZ) (60 mg/kg i.p.) was used to induce diabetes after 12 hr of fasting. Animals were considered diabetic with fasting blood glucose levels higher than 250 mg/dl. The rats were randomly divided into six groups with ten animals in each group (Table 1). Groups II-VI were diabetic cases. The treatments were started 72 hr after the STZ injection and after diabetes development. OLE (100, 200 or 400 mg/kg), and metformin (300 mg/kg) were administered orally using a gavage needle once a day for 6 weeks. Animals in groups I and II were treated with equal volume of normal saline and they served as vehicle control. After this treatment period, rats were subjected to passive avoidance learning test during days 37–42. Finally, all the animals were humanely scarified and the hippocampus and brain cortex were quickly removed and conserved at -80°C for oxidative stress assessment.

**Table 1:** Treatment protocols in different groups of rats

**Groups (n = 10)**	**Treatment protocols**
Group I (normal control)	Distilled water
Group II (diabetic control)	Distilled water
Group III (diabetes + Metformin)	Metformin (300 mg/kg body weight)
Group IV (diabetes + Extract 100)	OLE* (*100 mg/kg body weight)
Group V (diabetes + Extract 200) Group VI (diabetes + Extract 400)	OLE (200 mg/kg body weight) OLE (400 mg/kg body weight)

**Passive avoidance test (PA)**

PA task is based on negative tendency of the rats for a dark condition. The apparatus includes two matched-size compartments, which splits into a dark and an illuminated box with a guillotine gate that unites these two parts. Each rat was habituated to the box for two consecutive days before the training period (5 min per day). Each rat was placed in the lit up portion on the training day and the gate was freed after a habituation period (30 sec), and delay to enter the dark compartment was noted. After that, on the floor of the stainless steel structure, rats were exposed to 2 mA (50 Hz) foot shock for 2 sec, and then they were returned to their enclosures. In the memory retention phases, 3, 24, 48, and 72 hr after delivery of the foot shock, the rats entered the illuminated segment, and after the habitation time (30 sec), the door was opened and the time to reach the dark section was recorded. The total number of entries and time passed by the rats in the dark compartments were recorded (Salmani et al., 2018 ▶). 

**Biochemical assessments**

At the end of the PA experiment, the animals were anesthetized by ketamine and xylazine (10 mg/kg and 2 mg/kg respectively, i.p), blood samples were obtained and centrifuged at 3000 rpm for ten min, and serum was used to measure the blood glucose level. After that, the cortex and hippocampus were separated and washed by phosphate buffered solution (PBS). The serum, cortex and hippocampus were stored at -80°C until biochemical analysis. Samples were weighed and homogenized with PBS. The levels of MDA, total thiol (SH) content and the activities of CAT and SOD in both hippocampal and cortical tissues were measured.

**Serum glucose**

Fasting blood sugar (FBS) level was assayed by a commercial kit according to manufacturé s guideline (Pars Azmoon, Tehran, Iran). 

**Brain tissue oxidative stress assessment**

**Assessment of MDA**

Lipid peroxidation was determined in terms of the level of MDA. To measure lipid peroxidation, in a solution of 2 ml hydrochloric acid (HCl), thiobarbituric acid (TBA) and trichloroacetic acid (TCA), 1 ml of homogenous samples was added. The blend was kept in a hot water bath for 45 min. After reaching the room temperature, the mixture was centrifuged for 10 min and then absorption was read at 535 nm. The MDA level was estimated using a formula that was previously described (Farrokhi et al., 2014 ▶; Khodabandehloo et al., 2013 ▶). 

**Assessment of total thiol content**

The amounts of thiol content have been calculated by the Ellman method (Beheshti et al., 2017 ▶; Ellman, 1959 ▶; Mahmoudabady et al., 2017 ▶). 5, 5′-dithiobis (2-nitrobenzoic acid) (DTNB) was considered to measure total SH content in cortex and hippocampus tissues. The SH group reacts with DTNB and creates a yellow color complex. Here, 1 ml of mixture containing tris and ethylenediaminetetraacetic acid (EDTA) buffer (pH 8.6) and 50 µl of tissues homogenate were mixed and solution absorbance against Tris-EDTA buffer alone (A1) was assessed at 412 nm. Also, 20 μl DTNB solution was poured into A1 and 15 min was deposited at the room temperature, then the absorption was read again (A2). DTNB reagent absorption alone was considered the blank (B). Total thiol content (mM) = (A2-A1-B) × 1.07 / (0.05 × 14,150)

**Determination of anti-oxidant enzymes activity**

CAT activity was assessed (Aebi, 1984 ▶) according to the method that was mentioned in our previous report (Abareshi et al., 2016 ▶). A method defined by Balasubramanian and Madesh was used to measure SOD activity in brain tissues (Madesh and Balasubramanian, 1998 ▶).

**Statistical analysis**

All results are reported as mean±standard error of the mean (SEM) and were evaluated by SPSS 20. Analysis of all results was done using one-way ANOVA followed by a multiple comparison Tukey test. The difference was considered significant at p<0.05.

## Results

**The effects of OLE on passive avoidance test**

**Figure 1:**
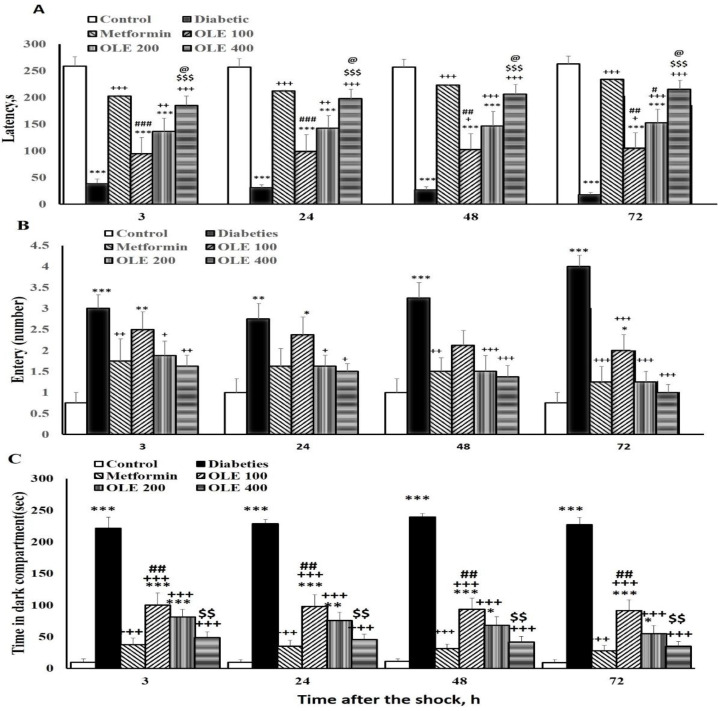
Comparison of the latency of the first entrance (A), the number of entrance (B) and time spent in the dark compartment (C), 3, 24, 48 and 48 hr after the shock in the study groups. Data are shown as mean±SEM (for each group n=8-10). *p<0.05, **p<0.01, and ***p<0.001 vs. the control group, +p<0.05, ++p<0.01, and +++p<0.001 vs. the diabetic group, #p<0.05, ##p<0.01 and ###p<0.001 vs the metformin group, $$p<0.01 and $$$p<0.001 vs. the OLE 100 group, @p<0.05 vs. the OLE 200 group

The PA test was carried out 3, 24, 48, and 72 hr after the shock was received. Compared to the control group, the diabetic rats had lower latency to go to the dark chamber in the retention tests (p<0.001). Metformin-treated ones displayed an improvement in latency to go into the dark chamber at all time-points (3, 24, 48, and 72 hr) after the shock, compared to the diabetic group (p<0.001). In three groups of OLE, the delay to enter the dark chamber was raised at various time points after the shock (p<0.05–p<0.001). There was no significant variation between OLE200 and OLE400 groups and the metformin group at either time point after the shock, while in the OLE100 group this latency was lower than the metformin group at either time point (p<0.01–p<0.001). Treatment with the maximum dose of OLE also improved the latency to enter the dark chamber compared to the lowest dose of OLE (p<0.001) and medium dose (p<0.05) at all the time points after the shock, Figure 1A.

The results indicated that the diabetic rats had more entries into the dark chamber compared to the control ones at all time-points of 3, 24, 48, and 72 hr after the shock (p<0.05–p<0.001). Metformin and all groups of OLE showed a reduced dark area entry 3 and 24 hr after the shock (p<0.05–p<0.001) in comparison with diabetic group. There was no significant difference between the impacts of the different doses of OLE and metformin as well as among the applied OLE doses (Figure 1B).

Data revealed that spending time in the dark compartment in the diabetic ones was greater than control groups 3, 24, 48 and 72 hr after the shock (p<0.001 for all). Compared to the diabetic group, metformin and all OLE groups spent less time in the dark compartment (p<0.001 for all).  However, this parameter was longer in the OLE100 ones in comparison with the metformin group (p<0.01). In the highest dose of OLE treated group, this spent time was less than the OLE100 ones (p<0.01, Figure 1C).

**Figure 2:**
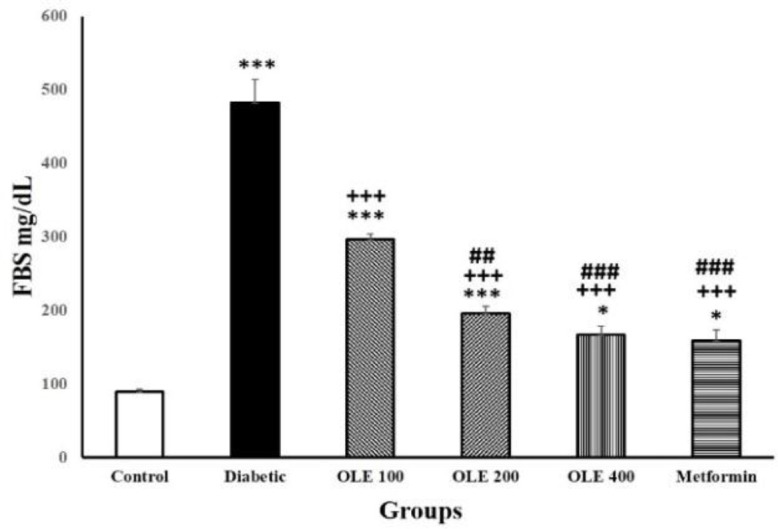
FBS levels in the serum of six study groups. Values are expressed as mean±SEM (n=8–10 in each group). *p<0.05 and ***p<0.001 vs. control group, +++p<0.001 vs. the diabetic group, ##p<0.01, and ###p<0.001 vs. the OLE 100 group

**The impact of OLE on serum blood glucose**

In the serum of diabetic groups, FBS levels increased dramatically compared to the control group (p<0.001). All OLE and metformin groups had significant reductions in FBS compared to the diabetic group (p<0.001). However, the FBS level in animals treated by OLE was greater than the control group (p<0.05–p<0.001). 

Moreover, the hypoglycemic effect of OLE200, OLE400 and metformin was comparable to that of OLE100, (p<0.01–p<0.001, Figure 2).

**The effects of OLE on oxidative stress state** **of the hippocampus**

**Figure 3:**
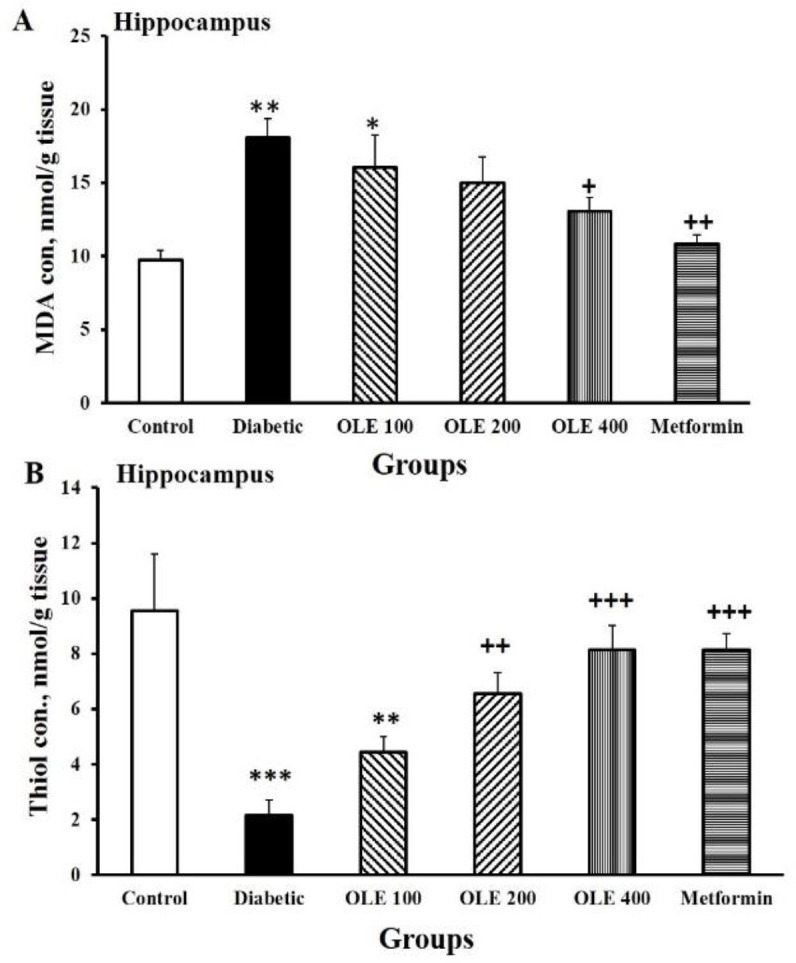
Comparison of MDA concentrations (A) and thiol content levels (B) in the hippocampal tissues among the six study groups. Data are presented as mean±SEM (n=8–10 in each group). *p<0.05, **p<0.01, and ***p<0.001 vs. the control group, +p<0.05, ++p<0.01 and +++p<0.001 vs. the diabetic group

In the hippocampus tissue, the MDA level in the diabetic rats was greater than the control group (p<0.01). Animals receiving the maximum dose of OLE and metformin showed a considerable decrease in MDA level in hippocampal tissue (p<0.05–p<0.001) compared to the diabetic group (Figure 3A). 

**Figure 4:**
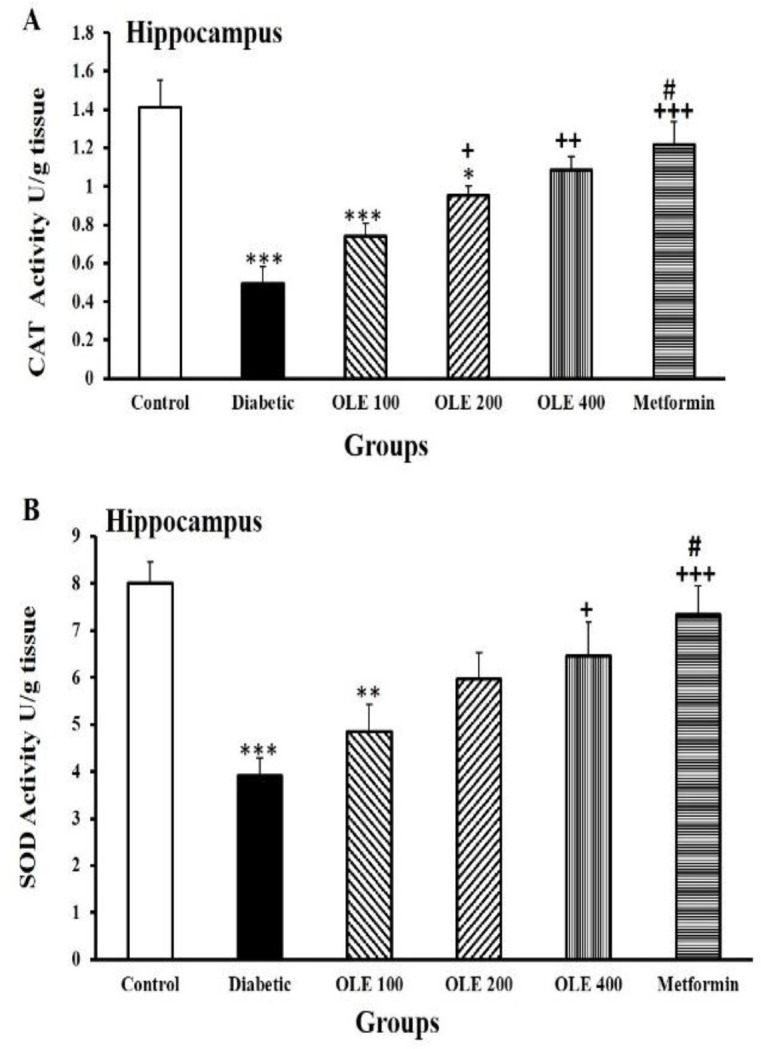
Comparison of CAT (A) and SOD (B) activities in the hippocampal tissues among the six study groups. Values are presented as mean±SEM (n=8–10 in each group). *p<0.05, **p<0.01 and ***p<0.001 vs. the control group, +p<0.05, ++p<0.01 and +++p<0.001 vs. the diabetic group, #p<0.05 vs. the OLE 100 group

Diabetes decreased the total content of thiol in hippocampal tissue (p<0.001) compared to the control group. In comparison with the diabetic ones, the amount of thiol in the hippocampus increased in the OLE 200, OLE400 and metformin groups (p<0.05–p<0.001). However, this augmentation did not show noticeable variations among all intervention groups, Figure 3B. In the hippocampal tissue of diabetes group, a considerable reduction of CAT activity was observed in comparison to the control ones (p<0.001). Animals receiving medium and maximum doses of OLE and metformin represented an increase in CAT activity compared to the diabetic ones (p<0.05–p<0.001). There were no significant differences among the three OLE-treated groups (Figure 4A).

In the diabetic group, SOD activity was considerably reduced in the hippocampal tissue compared to the control ones (p<0.001). Treatment with the maximum dose of OLE and metformin reversed the negative impacts of diabetes on SOD activity (p<0.05 and p<0.001, respectively). On the other hand, the increase of SOD activity in these groups was not comparable to the control group. There were noticeable differences in SOD activity levels between the lowest dose of OLE and metformin group (p<0.05, Figure 4B). 

**The impacts of OLE on oxidative stress state** **of cortical tissues**

**Figure 5:**
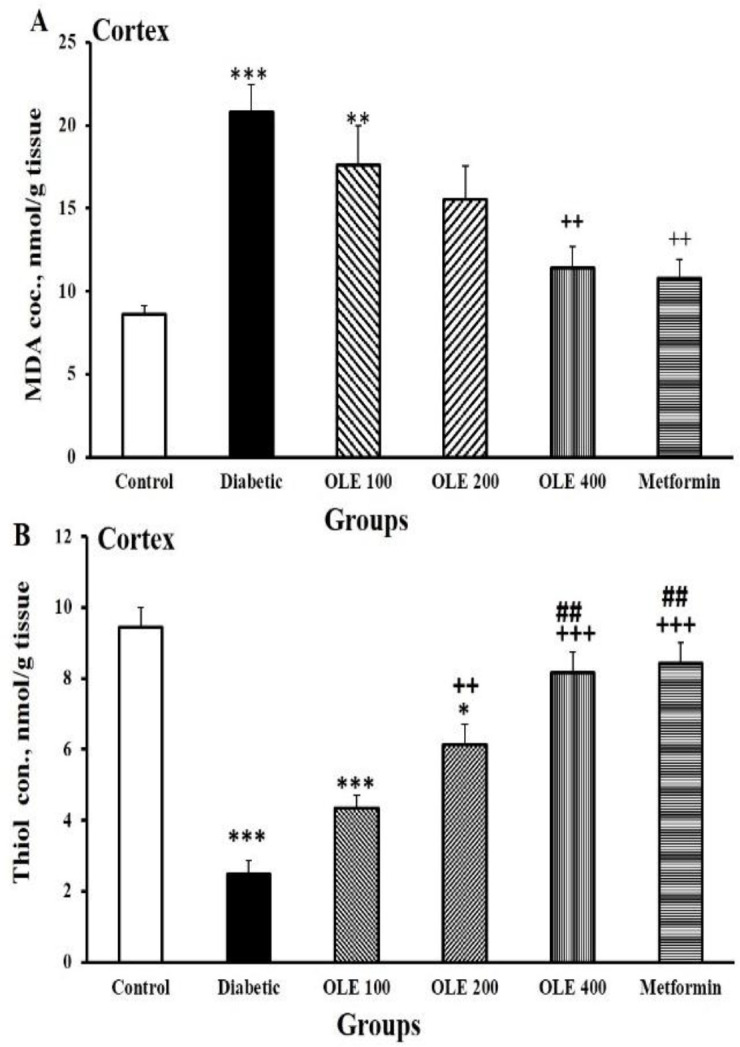
Comparison of MDA concentration (A) and total thiol content (B) in the cortical tissues of six study groups. Data are expressed as mean±SEM (n=8–10 in each group). *p<0.05, **p<0.01 and ***p<0.001 vs. the control group, ++p<0.01, and +++p<0.001 vs. the diabetic group, ## p<0.01 and vs. the OLE 100 group

Results revealed that diabetes enhanced MDA levels in cortical tissue compared to the control group (p<0.01). MDA concentrations were lower in the cortical tissues of the OLE 400 and metformin groups in comparison with the diabetic rats (p<0.01). There were no significant differences among all intervention groups (Figure 5A). 

Compared to the control ones, the cortical thiol concentration in the diabetic rats was considerably lower (p<0.001). OLE 200, OLE400 and metformin groups showed an increase in total thiol content in cortical tissues compared to the diabetic ones (p<0.01–p<0.001). Additionally, there was a considerable variation between the increase of total thiol amount in OLE400 and metformin groups, and OLE100 ones (p<0.01, Figure 5B).

**Figure 6:**
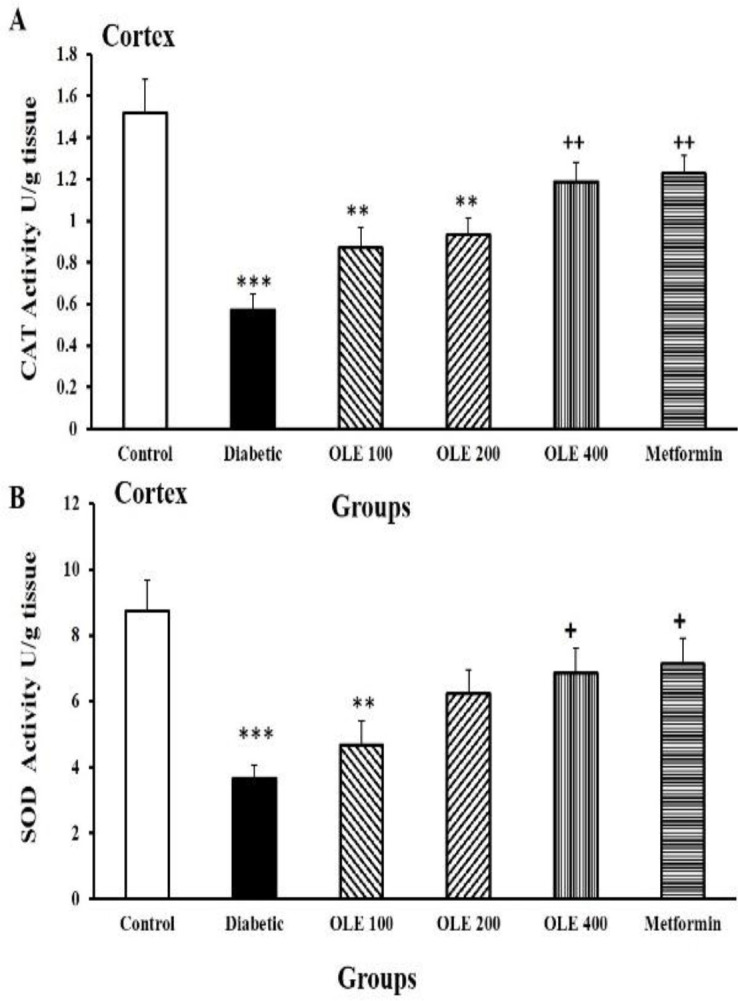
Comparison of CAT (A) and SOD (B) activities in the cortical tissues among the six study groups. Values are presented as mean±SEM (n=8–10 in each group). **p<0.01, and ***p<0.001 vs. the control group, +p<0.05, and ++p<0.01 vs. the diabetic group

Diabetes also resulted in a significant reduction in CAT activity in cortical tissue compared to the control group (p<0.001). OLE400 and metformin groups showed elevated CAT activities in the cortical tissue compared to the diabetic group (p<0.01). No significant differences were observed among different doses of OLE when compared to metformin-treated ones (Figure 6A).

SOD activity declined in the cortical tissues of the diabetic rats compared to the control group (p<0.001). Animals receiving OLE400 and metformin presented an enhancement in SOD activity compared to the diabetic group (p<0.05). There were no noticeable differences among all intervention groups (Figure 6B).

## Discussion

In the current study, our results demonstrated that chronic hyperglycemia in STZ-induced diabetes can lead to impaired learning and memory as well as biochemical consequences. Most of the negative impacts of chronic hyperglycemia on learning deficit were reversed by treatment with different doses of OLE in 6 weeks.

Previous research have shown that diabetes mellitus is linked to neurological problems such as weak learning and memory within the central nervous system (Abbasnezhad et al., 2015 ▶; Tuzcu and Baydas, 2006 ▶). The speed of data processing, working memory and certain aspects of attention were shown to reduce in patients with type 2 diabetes following acute hyperglycemia (Sommerfield et al., 2004 ▶). The results of PA task in our study, indicated fear memory impairment in diabetic rats. The changes and damages occurred in the hippocampal and cerebral cortex synaptic plasticity were displayed to contribute to the impairment of learning and memory (Fukui et al., 2002 ▶). In addition, clinical data showed that diabetes in people with diabetes is followed by learning and memory impairment (Biessels et al., 2006 ▶). Although the cause of these impairments is multifactorial, enough evidence is available regarding the role of increased oxidative stress in this situation. Following evaluating oxidative stress in diabetic animals, excessive production of ROS that results in cellular and oxidative damage in several areas of the brain including the hippocampus and cortex has been shown (Tuzcu and Baydas, 2006 ▶). These imbalances between oxidant and anti-oxidant agents are considered a possible mechanism for the complications, morphological abnormalities, and memory impairments in diabetes (Giacco and Brownlee, 2010 ▶). In our study, diabetic animals showed high levels of MDA in the cortex and hippocampus. The decreased activity of antioxidant enzymes is a cause for the increased lipid peroxidation in the course of diabetes. 

Here, we found that total thiol concentration, CAT and SOD activity levels were reduced in diabetic brain tissues. Our findings are in accordance with prior researches which suggest that during chronic diabetic neuropathy, antioxidant enzyme activities in the brain are reduced (Anwar and Meki, 2003 ▶; Baynes and Thorpe, 1999 ▶). Anti-oxidant compounds and free radical scavengers have been suggested to attenuate the complications of diabetes by suppressing generation of ROS during hyperglycemia (Al-Azzawie and Alhamdani, 2006a ▶). In the current report, it was found that metformin besides FBS reduction, ameliorated learning and memory impairment in the PA test in diabetic animals. Furthermore, metformin reversed the negative evidence of increased oxidative stress activity by lowering MDA level, elevating total thiol content, and improving the SOD and CAT activities. Several studies have shown the significant antioxidant effect of metformin in STZ-induced diabetic rats (Erejuwa et al., 2010 ▶; Liu et al., 2008 ▶; Majithiya and Balaraman, 2006 ▶). Thus, metformin in addition to its antihyperglycemic effect, plays a protective role against oxidative damage in the diabetic rats, proposing that it could be able to act as a beneficial neuroprotective agent in the course of DM (Tao et al., 2018 ▶).

As mentioned previously, of different compounds present in OLE, oleuropein is the most abundant phenolic constitute already recognized (Amro et al., 2002 ▶; Omar, 2010b ▶). Experimental investigations demonstrated the valuable impacts of oleuropein and its products such as hydroxytyrosol, for different biological processes, including scavenging of free radicals, and anti-hyperglycemic and neuroprotective impact. Oleuropein has been shown also to improve the levels of cognitive decline and memory impairment induced by colchicine in the hippocampus (Pourkhodadad et al., 2016 ▶). Diabetic rabbits treated with oleuropein showed a reduction in blood glucose levels along with declined MDA (Al-Azzawie and Alhamdani, 2006b ▶). These studies have highlighted that oleuropein may be helpful in preventing diabetic consequences associated with oxidative stress. In a model of heroin-induced brain injury, OLE was able to reduce MDA while activities of glutathione peroxidase, CAT and glutathione in OLE-treated groups were enhanced (Wang et al., 2013 ▶). OLE administration in diabetic animals indicated the beneficial impact of the treatment on fear memory deficiency by reducing the number of entry into dark chamber (Omar, 2010a ▶). In the current study, during the PA task, the numbers of entrance to the dark chamber were increased in the diabetic rats, indicating fear memory impairment. In addition, in these group of animals, latency to enter the dark chamber was reduced during the trial, and also an increase in time spent in the dark compartment was observed that could be corroborated memory retention impairment induced by DM. The biochemical and PA test values of the present study revealed that in diabetic rats, effects of higher doses of OLE were almost similar to metformin. Also, in the retention trial, the increased time spent in the dark apparatus and decreased latency to enter the dark compartment among diabetic rats were also inhibited by OLE treatment in a dose-dependent manner. As obviously indicated in our data, OLE in a dose-dependent manner not only exhibited preventive effects counteracting oxidative stress but also moderated cognitive decline and memory impairment caused by diabetes. Therefore, the presence of higher amount of phenolic compounds, with known antioxidant activity, in OLE can be attributed to more effectiveness of this extract.

The present data indicated that OLE besides its hypoglycemic effect, reduced MDA contents and on the other hand, ameliorated the total thiol content, and CAT and SOD activities in the hippocampal and cortical tissues.

Although there was no clear understanding of the specific mechanism of OLE to prevent learning and memory impairments but it is suggested that OLE might be utilized as a memory enhancer and neuroprotective agent and the beneficial benefits of the extract can be at least in part caused by prevention of oxidative damage in the brain tissues.

## Conflicts of interest

The authors have declared that there is no conflict of interest.
